# A simple polystyrene microfluidic device for sensitive and accurate SERS-based detection of infection by malaria parasites†

**DOI:** 10.1039/d3an00971h

**Published:** 2023-07-28

**Authors:** Maria João Oliveira, Soraia Caetano, Ana Dalot, Filipe Sabino, Tomás R. Calmeiro, Elvira Fortunato, Rodrigo Martins, Eulália Pereira, Miguel Prudêncio, Hugh J. Byrne, Ricardo Franco, Hugo Águas

**Affiliations:** a CENIMAT-i3N, Departamento de Ciência dos Materiais, Faculdade de Ciências e Tecnologia, FCT, Universidade Nova de Lisboa, and CEMOP/UNINOVA 2829-516 Caparica Portugal hma@fct.unl.pt; b Associate Laboratory i4HB – Institute for Health and Bioeconomy, Faculdade de Ciências e Tecnologia, Universidade NOVA de Lisboa 2829-516 Caparica Portugal ricardo.franco@fct.unl.pt; c UCIBIO – Applied Molecular Biosciences Unit, Departamento de Química, Faculdade de Ciências e Tecnologia, Universidade NOVA de Lisboa 2829-516 Caparica Portugal; d LAQV, REQUIMTE, Departamento de Química e Bioquímica, Faculdade de Ciências, Universidade do Porto Rua do Campo Alegre s/n 4169-007 Porto Portugal; e Instituto de Medicina Molecular João Lobo Antunes, Faculdade de Medicina, Universidade de Lisboa Av. Prof. Egas Moniz 1649-028 Lisbon Portugal; f FOCAS Research Institute, Technological University Dublin Camden Street Dublin 8 Ireland

## Abstract

Early and accurate detection of infection by pathogenic microorganisms, such as Plasmodium, the causative agent of malaria, is critical for clinical diagnosis and ultimately determines the patient's outcome. We have combined a polystyrene-based microfluidic device with an immunoassay which utilises Surface-Enhanced Raman Spectroscopy (SERS) to detect malaria. The method can be easily translated to a point-of-care testing format and shows excellent sensitivity and specificity, when compared to the gold standard for laboratorial detection of Plasmodium infections. The device can be fabricated in less than 30 min by direct patterning on shrinkable polystyrene sheets of adaptable three-dimensional microfluidic chips. To validate the microfluidic system, samples of *P. falciparum*-infected red blood cell cultures were used. The SERS-based immunoassay enabled the detection of 0.0012 ± 0.0001% parasitaemia in a *P. falciparum*-infected red blood cell culture supernatant, an ∼7-fold higher sensitivity than that attained by most rapid diagnostic tests. Our approach successfully overcomes the main challenges of the current *Plasmodium* detection methods, including increased reproducibility, sensitivity, and specificity. Furthermore, our system can be easily adapted for detection of other pathogens and has excellent properties for early diagnosis of infectious diseases, a decisive step towards lowering their high burden on healthcare systems worldwide.

## Abstract 2

Novel microfluidic SERS immunoassay to detect Plasmodium falciparum (Pf), with a LOD of 0.0012 ± 0.0001% parasitaemia, equivalent to ≈69 parasites per μL, a ∼7-fold higher sensitivity than most Malaria rapid diagnostic tests.

## Graphical abstract

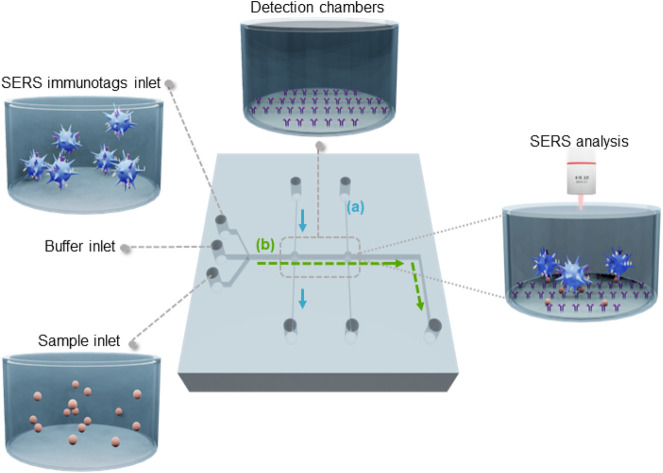

## Introduction

Despite significant efforts in the fight against malaria, the disease is still considered a major public health problem worldwide. 247 million new clinical episodes were estimated in 2021, which resulted in more than 600 000 deaths, 80% of which occurred in children under 5 years old.^1^ Inaccurate diagnosis is one of the causes of this high burden, along with increased drug resistance, climate change, and political and social factors.^2^

Malaria is caused by Plasmodium parasites, among which *P. falciparum* accounts for 90% of worldwide malaria mortality, especially in sub-Saharan Africa.^3^ Optical microscopy analysis of Giemsa-stained blood smears is the gold-standard technique for malaria diagnosis, but the need for easier assays with similar performance has led to the development of several antibody-based rapid diagnostic tests (RDTs).^4,5^ These are mostly lateral flow immunoassays, whose speed (<30 min), user-friendliness, and cost-effectiveness, make them applicable for widespread implementation at point-of-care testing (POCT).^6^ However, they still suffer from insufficient limits of detection (LOD recommended by the WHO is 200 parasites per μ L *i.e.* 0.004% parasitaemia), and high susceptibility to climate conditions.^6^ Other highly accurate diagnostic methods, such as fluorescence immunoassays, polymerase chain reaction (PCR) and its on-field version known as loop-mediated isothermal amplification (LAMP), require complicated operating procedures, long incubation times, and specialized equipment and staff.^7^

Microfluidics can address some of the limitations of existing detection methods by increasing sensitivity in an automated and portable analysis system. Lee *et al.*^8^ used the high surface-to-volume ratio provided by the reduced size of the Optimiser™ microfluidic chip^9^ to improve the conventional enzyme linked immunosorbent assay (ELISA).

One of the disadvantages of conventional silicon or PDMS microfluidic chips is their fabrication and optimisation processes, which require cleanroom facilities, and laborious and expensive fabrication protocols.^10,11^ Several alternative methods have been proposed to reduce the complexity and cost of microfluidics microfabrication techniques, such as microwire moulding,^12^ 3D printing,^10,13^ and laser machining,^14,15^ among others.

In the present work, CO_2_ laser machining was selected for the fabrication of the microfluidics device. This technique is especially suited for low heat conductive materials, such as polymers.^14,15^ Its low-cost, high speed, and contactless features have been demonstrated in paper,^16^ poly(methyl methacrylate) (PMMA),^17^ polycarbonate,^18^ and polystyrene (PS).^15,19,20^ Despite its excellent performance, the use of this technique in biomolecular detection platforms remains very limited. Hu *et al.*^15^ developed a microfluidic chip for protein digestion, and Oliveira *et al.*^21^ fabricated a droplet digital loop-mediated isothermal amplification (LAMP) chip. These reports underline the shrinking properties of biaxially oriented PS thermoplastic sheets for developing new biosensors.

Surface enhanced Raman scattering (SERS) offers high sensitivity and multiplex capability for detection of several biomarkers.^22,23^ The advantages of combining the high sensitivity and specificity of SERS with microfluidics for *Plasmodium* detection were shown by Chen *et al.*^24^ with a SERS lab-on-chip device with a sensitivity of 0.0025% parasitaemia. This assay was performed using haemozoin as a biomarker, which does not allow the distinction between viable parasites and remains from a prior infection. To enable differentiating between active and prior infection, we selected *P. falciparum* histidine-rich protein 2 (*Pf*HRP2) and *P. falciparum* lactate dehydrogenase (*Pf*LDH)^25^ as parasite biomarkers. Several studies have shown a correlation between plasma *Pf*HRP2 levels with the disease severity.^26^ However, *Pf*HRP2 is not detected in cases of non-*falciparum* malaria and *pf*hrp2/*pf*hrp3 deletions.^27^ On the other hand, *Pf*LDH shares epitopes with other *Plasmodium* species, enabling its use as a parasite species-unspecific biomarker, and may therefore aid in cases of malaria misdiagnosis.^28^*Pf*LDH, is the most expressed enzyme required for anaerobic adenosine triphosphate (ATP) generation in *P. falciparum* and is produced by sexual and asexual stages of the parasite. Due to its function, *Pf*LDH is indicative of a recent infection, in contrast to *Pf*HRP2 that remains detectable for 1–5 weeks after treatment. As a result, the combined use of both biomarkers offers enhanced reliability in the detection of active infections.^28^

Our approach relies on the use of “SERS-tags” containing (i) metal nanoparticles for enhancement of the Raman signal of (ii) Raman reporters, and (iii) antibodies against the selected biomarkers as the biorecognition elements.^42^ As recently shown by us,^23,42^ SERS immunotags can be integrated with the direct classical least squares (CLS) method for improved LOD and efficiency of SERS duplex detection. The novel microfluidic device is fully transparent, inexpensive and can be rapidly produced. It has a remarkable design flexibility, which brings the microfluidic SERS-based assays technology several advantages relative to currently available assays. A fully functional SERS-based microfluidic system is demonstrated, and its applicability is shown by the detection of two *P. falciparum* antigens, *Pf*HRP2 and *Pf*LDH, enabling simple, rapid, and accurate diagnosis of malaria.

## Results and discussion

### SERS-based immunoassay microfluidic chip design

**Fig. 1:**
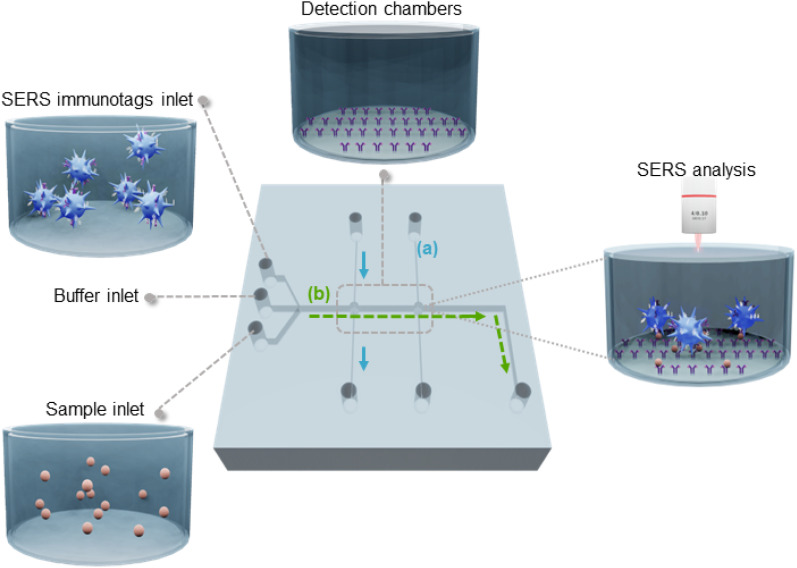
Flow-through microfluidic device integrated with TO-RCH for the SERS-based immunoassay. (a) Detection chambers with TO-RCH membranes are functionalised with antibodies to function as capture platforms – blue arrows indicate the flow direction. (b) The sample containing target biomarkers is introduced through the sample inlet, which are captured by the antibodies in the detection chambers. SERS immunotags flow through the detection chamber to form the sandwich complex – the green arrows indicate the flow direction. As the final step, the SERS signal from the complex is measured and analysed through a confocal Raman microscope. The presence of two detection chambers enables the functionalization of the capture platform with different antibodies, the first one with the test antibody and the second with a control antibody. The flow rate and total reaction time are defined by capillary pumps. The area of the microfluidic chip is 3 × 3 cm^2^, a channel of 200 μm and both detection chambers have a 500 μm radius.

The design of the microfluidic device is shown in Fig. 1. Its mode of operation relies on the use of two chambers, one for detection of both *Plasmodium* biomarkers (detection chamber, Fig. 1, left) and the other to detect the control SERS-tags (control chamber, Fig. 1, right), similar to a typical lateral flow immunoassay.^25^ To obtain an efficient immobilisation of the capture antibodies in the chambers, both are coated with TO-RCH to which the capture antibodies are covalently linked, as previously described.^23^

The microfluidic device also includes two perpendicular sets of channels: one for sensor fabrication (Fig. 1(a) blue), the other for sample analysis (Fig. 1(b) green). The set of channels for sensor fabrication consists of two independent parallel channels, each leading to one of the chambers. This design choice allows parallelisation of fabrication into one single working unit, keeping the detection chambers spatially separated to clearly distinguish them. Immobilisation of the respective antibodies into each detection chamber is performed in parallel channels, avoiding cross-contamination. In addition, the perpendicular configuration of the channels for fabrication and the channel for the assay ensures that the capture antibodies are confined in the detection chambers and that, while performing the assay, no reaction occurs outside these areas.

The assay starts by filling the channels and chambers with buffer through the buffer inlet (Fig. 1). The sample is then introduced in the sample inlet and pumped through the channel (b) into the detection chamber, where the biomarkers in the sample (target antigens) bind to the capture antibodies. Finally, SERS tags are injected to conclude the sandwich type immunoassay.^42^ In the case of positive samples, SERS tags are captured in both chambers, whereas for a negative sample, SERS tags are only captured at the control chamber. The microfluidic chip is then placed in the Raman microscope for the SERS analysis of the chambers.

### Fabrication and characterization of the microfluidic device

**Fig. 2:**
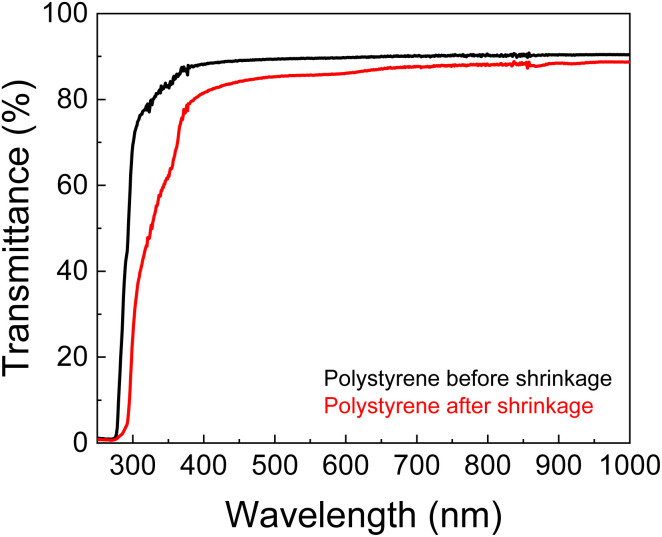
Total transmittance UV-Vis-NIR spectra of PS sheets, before and after shrinkage process. The presented spectra are the median of three independent measures.

One of the striking features of PS for SERS applications is it is transparency, which allows Raman light to pass through. This behaviour was evidenced in the UV-Vis-Near-infrared spectra of polystyrene, presenting typical high transparency for wavelengths in the visible and NIR range (Fig. 2).^29^ The transmittance at 633 nm from a single sheet of unshrunk PS is ≈90% and this value decreases by only 3% after the shrinkage process. This proves that PS is suitable for creating transparent chambers allowing further on-chip and real-time SERS analysis using the 633 nm laser as source. Thus, the detection chamber is enclosed by a single sheet of PS, to ensure transparency and to minimise possible optical artifacts in SERS measurements.

**Fig. 3:**
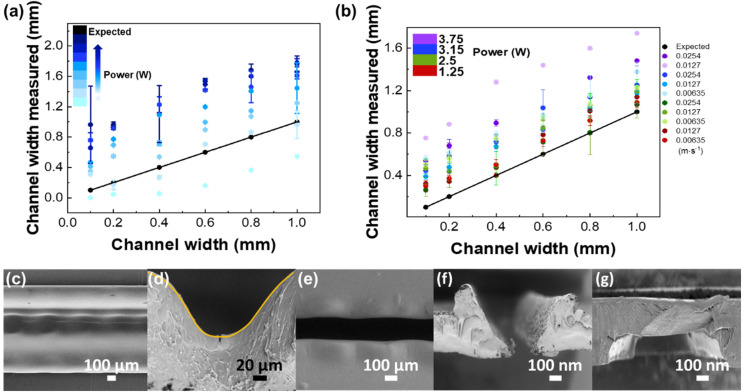
CO_2_ laser resolution tests comparing the pattern from the digital drawing to that produced by the laser. Comparison between imposed channel width and channel width produced by ablation; the black line represents the ideal values *i.e.* imposed and produced channels widths have the same values. (a) Channel width produced by laser ablation with different powers (1.25 to 10 W) at constant velocity (0.0381 m s^−1^). (b) Channel width produced by laser ablation with different powers (1.25 to 10 W) and different velocities (0.00635–0.0508 m s^−1^). Error bars correspond to the standard deviation from ten independent measures. SEM images of the patterned microchannel when the laser ablation process is not effective to cut the channel: (c) top view and (d) cross-section view, showing a channel of width ≈1000 μm and depth ≈100 μm. (e) SEM image of the top view of the patterned microchannel after the laser ablation process (width ≈180 μm). The cross-section views of a microchannel (width ≈180 nm and depth ≈600 μm) (f) after the photoablation process and (g) after the thermal bonding (width ≈900 μm and depth ≈200 μm).

Besides the thermal diffusivity and composition of the material, the microchannel patterning depends on the intensity distribution of the laser beam (being focused or unfocused),^17^ the laser power, and cutting speed.^30^ Thus, to pattern a microfluidic channel with the desired width, two patterning parameters were varied – velocity and power. In each case, the laser beam was focused on the PS sheet to obtain a narrow and precise ablation.^17^ The best resolution was assessed by patterning lines with widths from 0.1 to 1 mm. As shown in Fig. 3a and b, the photoablation process always produced broader channels than expected from the digital drawing, with higher standard deviations for higher applied laser power.

At low laser power (1.25 W) and high velocities (0.0508 m s^−1^), the laser did not effectively cut the channel. As can be seen in the SEM micrographs (Fig. 3c and d), the resulting channels are clearly corrugated (Fig. 3c, top view), because of the pulsed character of the laser beam, which leaves a smooth and curvilinear engraved surface (in a Gaussian shape) (Fig. 3d, cross-section), as expected.^30^ This behaviour is due to the high thermal conductivity of PS (0.033 W m^−1^ K^−1^) that leads to an effective diffusion of heat and homogenous ablation in all directions. Conversely, the use of higher laser power (≥10 W) and lower speeds (≥0.00635 m s^−1^) leads to degradation of the pattern by accumulation of material on pattern edges, due to the higher diffusion times. The smallest standard deviation obtained was for a laser power of 2.5 W and velocities of 0.0381 and 0.0254 m s^−1^ (Fig. 3c, b and e), representing a good balance between patterning quality and degradation.

Following the patterning resolution test, a channel of 1 mm width was repeated 10 times and subsequently shrunk in an oven at 155 °C, for 5 min. To obtain a uniform flat surface instead of Gaussian distribution generated by the laser engraving, a double line design, with a 200 μm line spacing, was used. The dimensions of the microchannel obtained for 2.5 W laser power and 0.0254 m s^−1^ velocity were 239 ± 2 μm height (corresponding to the thickness of a single PS sheet) and 230 ± 23 μm width, with a relative standard-deviation (RSD) of 10.2%, indicating a good reproducibility. After patterning, it is possible to observe a Gaussian-like cross-section profile of the microchannel followed by small bumps in the edges of the microchannel (Fig. 3f), resulting from the surface tension gradient of the melted polystyrene.^31^ After the cover plate was bonded with the middle layer, by heating in the thermal press, the bumps disappeared, and no deformation of the channel was observed (Fig. 3g).

**Fig. 4:**
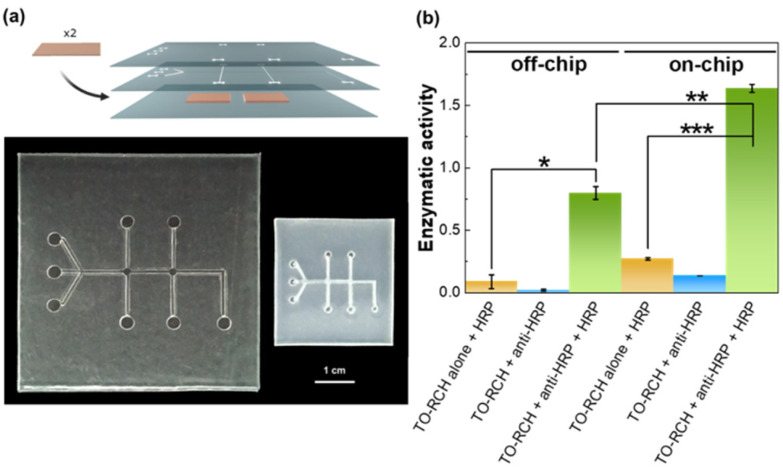
(a) Top: schematic representation of the 3D multilayer chip (TO-RCH is represented in orange just for visualisation purposes, the membrane is transparent as previously mentioned). Bottom: top perspective of the PS microfluidic chip. 3D multilayer chip with microchannels patterning after photoablation and thermal bonding (left, 6 × 6 cm^2^); and the same chip after shrinking (right, 3 × 3 cm^2^). (b) Performance of the PS-based microfluidic device evaluation by peroxidase enzymatic activity. The enzymatic activities for “off-chip” were measured in a TO-RCH membrane outside the chip, whereas the results “on-chip” are for the microfluidic device; yellow bars: control with addition of HRP only and washing; blue bars: control with anti-HRP immobilised, but no HRP addition; green bars: enzymatic activity detected after capture of HRP by immobilized anti-HRP antibody. One-way analysis of variance (ANOVA) was applied for statistical comparison, followed by the Tukey's multiple comparison test, *p ≤ 0.005, **p ≤ 0.0001, ***p ≤ 0.00001.

The device was designed in a 3D multi-layered system that can be easily adjusted for other applications. The device is composed of three stacked layers (Fig. 4a): the top layer was patterned with the inlets and outlets only; the middle layer, used for the immunoassay, is patterned with the channels, chambers and inlets and outlets; the bottom layer is a flat non-patterned PS sheet, to seal the middle layer. After the photoablation process, all the layers were stacked, including the TO-RCH membrane for antibody immobilisation between the middle and bottom layer. Binding between layers was performed by thermal pressing at 110 °C which is 3 °C higher than the glass transition temperature, for 20 min. This temperature allows not only to firmly bond the layers but also to obtain a smooth surface inside the microchannels.

The final PS chip was obtained by shrinkage in an oven at 155 °C, for 5 min. To avoid inhomogeneous shrinking, the aspect ratio of the chip was always 1. The shrinkage ultimately results in a thickness increase (63%), as expected for this type of thermoplastics.^20^ The final dimensions of the chip were ≈3 × 3 cm^2^ with microchannels of 129 ± 8 μm width and 998 ± 53 μm height, and a detection chamber of 559 ± 10 μm, yielding a miniaturisation of ≈58% (Fig. 4a). Shrinkage decreases the transparency, as can be seen in Fig. 4a, and expected from the results of Fig. 2, but since the detection chamber is below a single sheet of PS, the transmittance of ≈87% (see Fig. 2) is still very appropriate for the Raman measurements.

The surface roughness of the channels is related to the hydrophilicity, which is an important parameter when developing a device for biodiagnostic applications. The more hydrophobic a material is, the higher the adsorption of small hydrophobic molecules onto the channel walls.^32,33^ As a result, the channels with a higher roughness would retain more non-specific molecules and consequently, increase the probability of non-specific binding of the immunotags ultimately compromising the detection of the SERS immunotags. Thus, higher roughness can be correlated with a lower assay sensitivity. To ensure that the microchannels had minimal protein adsorption, their hydrophilicity was evaluated by measuring water contact angles and AFM (section S3 in the ESI†). Both techniques showed that, in the fabrication process, the hydrophilicity of the surface increases, possibly due to the decrease of surface roughness.

The viability of the PS microfluidic chip for immunoassays was assessed using a peroxidase (HRP)/anti-peroxidase antibody system, a method which has proven successful for assessing the functionality of biosensors.^23^ First, anti-HRP antibody was immobilised onto the TO-RCH membrane inside the chip (termed as “on-chip”) and onto a TO-RCH membrane treated at 110 °C, for 20 min, and 155 °C for a further 5 min, to simulate the fabrication process (termed “off-chip”). After addition of HRP and washing, the enzymatic activity was measured, and the results are shown in Fig. 4b. “On-chip” enzymatic activity is approximately twice “off-chip” enzymatic activity (Fig. 4b, green bars). This doubling of activity “on-chip” highlights the advantages of performing the biochemical reaction inside the microfluidic device, where the micrometre scale maximises mass transport.^34^

Additionally, all the controls performed either on or off-chip (Fig. 4b, yellow and blue bars) showed a much smaller enzymatic activity. In particular, the controls with HRP alone (Fig. 4b, yellow bars) show that the amount of active HRP adsorbed in the absence of anti-HRP is minimal. These results indicate a successful detection of antigen–antibody complex presence, thus confirming the viability of this PS microfluidic device for immunoassays application.

### Performance of the microfluidic device for *Pf*HRP2 detection and quantification

The ability of the chip to detect *Pf*HRP2 was assessed using recombinant *Pf*HRP2 spiked to the supernatant of red blood cells (RBCs) and using native *Pf*HRP2 in the supernatant of infected red blood cells, to confirm that a possible variation of *Pf*HRP2 would not compromise the reactivity of the immunoassay. Details about the preparation and characterisation of recombinant *Pf*HRP2, as well as reactivity studies with anti-*Pf*HRP2 monoclonal antibody can be found in section S1 of the ESI.†

**Fig. 5:**
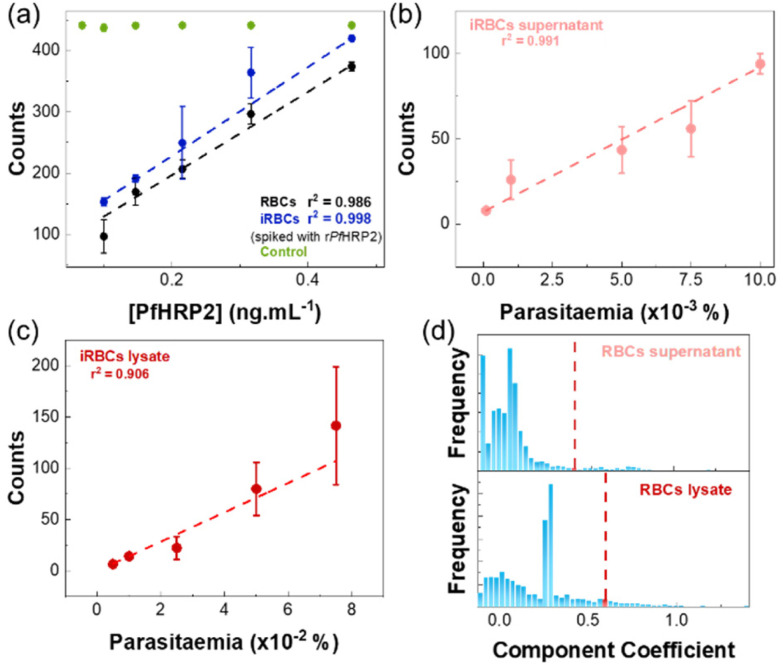
Results of the SERS immunoassay on the microfluidic device. Error bars indicate the standard deviation of three independent experiments. (a) Linear fit and corresponding SERS counts from the immunoassay of supernatants of RBCs and iRBCs spiked with recombinant *Pf*HRP2, obtained by the CLS method: black points and line: RBCs; blue points and lines iRBCs; green points: signal from the control chamber. (b) Linear fit and corresponding SERS counts from the immunoassay of samples of supernatant of iRBCs cultures. (c) Linear fit and corresponding SERS counts from the immunoassay of samples of lysates of iRBCs cultures. (d) Histogram of component coefficient frequencies for blanks in the SERS immunoassay (top: RBCs lysate and bottom: RBCs supernatant). The red dashed line represents the 0.452 and 0.627 component coefficient that was considered as the threshold for the positive detection of a SERS immunoassay in RBCs supernatant and RBCs lysate, respectively.

After preparing the chip with capture antibodies, samples were inserted through the microfluidic device and incubated for 10 min at 25 °C. The microchannels were then washed with PBS-T buffer, before inserting the SERS immunotags for the formation of the SERS active immunocomplex.^42^ The final wash with PBS-T eliminates the excess of SERS immunotags, leaving the sandwich complex to be detected using a micro-Raman spectrometer. Results are presented in Fig. 5 and Table 1.

**Table 1:** Sensitivity of the SERS immunoassay on the microfluidic device on the supernatant samples of RBCs and iRBCs – spiked and non-spiked with recombinant *Pf*HRP2. LOD and LOQ were calculated by the standard IUPAC method^38^

Type of sample	Spiking	LOD	LOQ
RBCs supernatant	Recombinant *Pf*HRP2	15.3 ± 0.2 pg mL^−1^	73 ± 1 pg mL^−1^
iRBCs supernatant	Recombinant *Pf*HRP2	14.2 ± 0.5 pg mL^−1^	68.0 ± 0.2 pg mL^−1^

iRBCs supernatant	—	0.0012 ± 0.0001%	0.0058 ± 0.0006%
iRBCs lysate	—	0.013 ± 0.005%	0.06 ± 0.02%

Fig. 5a shows the results obtained in the quantification of samples of RBCs and iRBCs supernatants spiked with recombinant *Pf*HRP2. The response was similar for both matrixes, with a limit of detection (LOD) of 15.3 ± 0.2 pg mL^−1^ and 14.2 ± 0.5 pg mL^−1^ in RBCs and iRBCs matrixes, respectively (Table 1). These values are well below the LODs of other detection methods (Table S2 in the ESI†). In both cases, a linear response was detected up to 15 pg mL^−1^. In all cases, SERS signals of the control were similar over the range of *Pf*HRP2 concentrations (RSD of 0.72%), validating the results of the assay.

To assess the feasibility of using the chip to quantify parasitaemia levels, iRBCs culture samples were used. SDS-PAGE and western blot analysis (described in detail in section S1.3 of the ESI†) applied to supernatants and lysed iRBCs allowed to assess the presence of native *Pf*HRP2 in both fractions which agrees with observations by other authors.^35^ As reference, parasitaemia levels of these samples were measured in thin blood smears using Giemsa stain on microscope slides. Measurements were performed using supernatants and lysates of iRBCs culture samples, and results are shown in Fig. 5b and c, respectively. In both cases, a linear response was observed, but the LOD for iRBCs lysates was approximately 10 times higher than for iRBCs supernatants, which is a consequence of the higher CLS threshold observed for the iRBCs. This behaviour has been previously observed^36^ and it was related to high non-specific binding, due to the presence of the cell contents in the lysates.

For supernatants of iRBCs culture samples, a linear response was obtained for parasitaemia ranging 0.0001–0.0025% (6–1453 parasites per μL; Fig. 5b), with a LOD of 0.0012 ± 0.0001% (≈69 parasites per μL) (Table 1). This LOD is below the threshold recommended by the WHO of 200 parasites per μL, and seven-fold lower than the minimum level usually attained by most RDTs recommended by the WHO.^1,37^ Thus, the microfluidic SERS immunoassay fabricated in this work shows a promising approach to bring a fast, easy, and cost-effective platform for malaria diagnosis.

Table S2 in section S5 of ESI† summarises and compares the sensitivity of published malaria detection methods, with our system. The performance of the microfluidic based biosensor device described here is superior to that of ELISA,^8^ other microfluidic devices,^39^ and SERS immunoassays.^40^ It is also comparable to methods based on hemozoin detection,^41^ which present important limitations, as described before in the “Introduction” section.

It should be noted that the sensitivity obtained in this work is closely related with the produced SERS immunotags. In this work, we used AuNSs not only for signal enhancement, but also as a structural scaffold. The advantage of using AuNSs is the sharp branches emanating from a core, giving several intrinsic hotspots per particle with multiple resonances – the so-called “sharp tip effect”.^42–44^ As a result, the AuNSs provide plasmonic near-field enhancements and a lightning rod effect (maximised in a tip-to-tip nanostar dimer) which leads to enhancement factors of orders of 10^9^. These AuNSs were functionalised and conjugated with the appropriate amounts of the MBA and antibody to AuNSs, as previously developed by us^42^ resulting in the formation of highly active stable bioconjugates.

Additionally, the microfluidic SERS immunoassay presented here is easier to fabricate than alternative designs.^39,40^ Hence, the proposed methodology is based on a Raman confocal microscope, whereby the antibody capture platform within the detection chamber acts as the sample, which provides an enhanced response through the combination of SERS tags and nanoparticles. Furthermore, the measurements were performed by using spatial maps which allow several measurements within the same sample, contributing to the accuracy of the assay. This sensitivity can be further increased if the SERS immunotags are developed to have the fragment antigen-binding (Fab) exposed using other techniques (*e.g.*, photochemical immobilization technique).^45^

Thus, this represents an advantage in terms of the measured sensitivity and robustness, although in its current form it has limited applicability for field deployable applications, which would require a different kind of study and optimization towards that.

Nevertheless, the PS microfluidic SERS-based immunoassay presented herein can be applied in healthcare centres and consequently, alleviate the high burden on healthcare systems worldwide.

Section S5 of the ESI† also presents reproducibility and selectivity experiments, to determine the ability of the device to discriminate *Pf*HRP2 within a complex sample, as well as assessment of time and thermal stability of the microfluidic device's activity.

#### Duplex assay

**Fig. 6:**
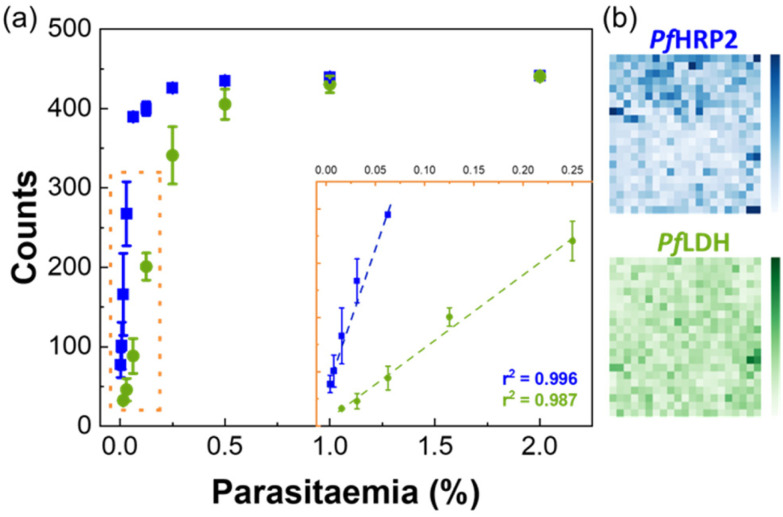
Duplex detection of *Pf*HRP2 and *Pf*LDH. (a) Linear fit and corresponding SERS counts from the microfluidic immunoassay, obtained by the CLS method. Blue data with the coefficient of determination of *r*^2^ = 99.6% corresponds to the data for *Pf*HRP2 and the green data with the coefficient of determination of *r*^2^ = 98.7% corresponds to the data for *Pf*LDH. Error bars indicate the standard deviation of three independent experiments. (b) Pixelated SERS maps for duplexing detection. The CLS method allowed data deconvolution generating the blue map (*Pf*HRP2 detection) and green (*Pf*LDH detection) components. Colour intensity is proportional to the CLS score.

Parasites with gene deletions for *Pf*HRP2 (*pfhrp2*) and *Pf*HRP3 (*pfhrp3*) are completely undetectable by most RDTs. Unfortunately, the predominance of these parasites has been documented in several areas including South America, Asia, Middle East, and Africa,^1^ which severely limits the efficacy of *Pf*HRP2-based RDTs. Additionally, *Pf*HRP2 can persist in the bloodstream after the disease has subsided.^25^ Therefore, to improve the usefulness of the proposed microfluidic system, a duplexed SERS immunoassay was performed. The TO-RCH platform was functionalised with two different antibodies (anti-*Pf*HRP2 and anti-*Pf*LDH) to capture their respective antigens. The immunocomplex for each antigen is accomplished by the same two antibodies conjugated with distinct Raman reporter molecules (MBA and DTNB) in SERS immunotags. The results are shown in Fig. 6. The CLS method was able to distinguish the two specific Raman reporters and the calculated CLS coefficients for MBA and DTNB were correlated with the parasitaemia achieving a LOD of 0.002% for *Pf*HRP2 and 0.007% for *Pf*LDH. The LODs obtained indicate that this system constitutes a promising approach for multiplex detection that could avoid false negative results due to genetic deletions or to non-*falciparum* malaria cases.

## Experimental

### Materials and methods

The following reagents were used: gold(iii) chloride solution 30 wt% gold in dilute hydrochloric acid (99.99%), sodium citrate tribasic dihydrate (99.0%), silver nitrate (99.9999%), tris(hydroxymethyl)-aminomethane, ethylenediamine tetraacetic acid, acetic acid (≥99%), ethanol; the Raman reporters, 4-mercaptobenzoic acid (MBA) and 5,5-dithio-bis-(2-nitrobenzoic acid) (DTNB); the crosslinking reagents, (1-ethyl-3-(3-(dimethylamino)propyl)carbodiimide – EDC and *N*-hydroxysulfosuccinimide – SNHS); buffers, (2-(*n*-morpholino)ethanesulfonic acid – MES, phosphate-buffered saline and potassium phosphate buffer), as well as the reagents used in regenerated cellulose-based hydrogel (RCH) platform preparation and in antibody immobilisation protocol, namely, lithium hydroxide (≤98%), microcrystalline cellulose (powder: 20 μm), glacial acetic acid, 2,2,6,6,-tetramethylpipelidine^−1^-oxyl radical, sodium bromide, sodium hypochlorite, bovine serum albumin, skim milk powder, Tween 20, horseradish peroxidase were all purchased from Sigma-Aldrich, St Louis, USA. The anti-peroxidase antibody was purchased from Antibodies-Online, Germany and the mouse monoclonal anti-*Pf*HRP2 and anti-*Pf*LDH antibodies were from Meridian Life Science, Luckenwalde, Germany and Gentaur, Kampenhout, Belgium, respectively. The immunopure mouse anti-IgG was purchased from ThermoFisher Scientific, Waltham, USA. Reagents used for obtaining *P. falciparum* culture supernatant, red blood cells (RBCs) infected red blood cells (iRBCs), and parasite lysate: Roswell Park Memorial Institute (RPMI) – 1640, albumax II, glucose, 4-(2-hydroxyethyl)^−1^-piperazineethanesulfonic acid (HEPES), sodium bicarbonate, gentamycin, hypoxanthine, and l-glutamine, were all purchased to GIBCO, Thermo Fisher Scientific, Waltham, USA. Urea (≥99.5%) was purchased from Carl Roth GmbH, Germany. Enzymatic assay reagents, 2,2′-azino-bis(3-ethylbenzothiazoline-6-sulfonic acid) was from Roche, France and hydrogen peroxide (30%) and nitric acid (65%) by Panreac AppliChem, Germany. l-Ascorbic acid (99.9%) was purchased from Fluka, Buchs, Switzerland. Protein determination was performed by the bicinchoninic acid method using a kit from Sigma-Aldrich, St Louis, USA. All chemicals were of the highest purity available. Ultrapure water (18.2 MΩ cm at 25 °C) was used for the preparation of all solutions, unless stated otherwise.

### Polystyrene chip fabrication

Fabrication of the PS chip was by patterning transparent PS sheets (Vaessen Creative shrink sheets, China) by CO_2_ laser ablation (VLS 3.50, 50 W, Universal Laser Systems, Scottsdale, AZ, USA) with a wavelength of 10.6 μm, a focal length of 50.8 mm, a beam diameter of 0.127 mm, and maximum power output of 50 W. To provide the smoothest channels, the repetition rate was set to the maximum, 1000 pulses per inch.^14,46^ This repetition rate was used through all the optimisation experiments. Adobe Illustrator (Adobe systems software, Ireland) was used to design the patterns on the PS sheets. The patterned PS sheets were cleaned in ultrasound with ethanol for 10 min, followed by 10 min in deionised water. This reduces the influence of other impurities on the hot bonding of plates. The sheets were then stacked together with the immobilisation surface, the (2,2,6,6-tetramethylpiperidn-1-yl)oxyl (TEMPO) oxidised RCH, (TO-RCH), and proceeded to thermal bonding. Thermal bonding was achieved by thermal pressing at 110 °C for 20 min. The bottom and upper sides of the device in contact with the hot side of the press were changed every 5 min to maintain both sides in contact with the hot side. The microfluidic chip fabrication process was completed by shrinking the PS bound sheets in a furnace (Nabertherm l3/11/B170, Nabertherm GmbH, Lilienthal, Germany) at 155 °C for 5 min. The final chip was washed with ultrapure water and stored in phosphate-buffered saline with Tween 20 (PBS-T) at 4 °C until further use. All the steps of the fabrication procedure are described in Fig. S1 in the ESI.†

The hydrophilicity of the microchannels, an important parameter to minimize adsorption of interfering hydrophobic molecules, was assessed during the laser ablation, thermal bonding, and shrinking processes (see section S4 in the ESI†).

### Preparation of biological samples

Procedures for expression, purification, and biochemical characterisation of recombinant *Pf*HRP2 were previously described,^23^ and are further detailed in section S1.1 of the ESI.†

Procedures used to obtain the *P. falciparum* culture supernatant, infected RBCs, and parasite lysates for the SERS-based microfluidic immunoassay, were as described before,^47^ with modifications detailed in section S1 of the ESI.†

### SERS-based immunoassay of *Pf*HRP2 inside the microfluidic device

The SERS-based immunoassay of *Pf*HRP2 using the regenerated cellulose hydrogel (TO-RCH) was performed as previously described.^23^ Detailed information is presented in section S2 of the ESI.† Polyethylene tubes and syringes were washed with ethanol, and, together with the chip, were pre-blocked with PBS-T. MES 10 mM pH 6.5, which was inserted at 0.5 μL min^−1^ for 1 h at room temperature (pumps Legato 210P and Legato 210, KD Scientific). For antibody immobilisation, 25 μL EDC and SNHS at 20.86 mM and 50.66 mM, respectively, were inserted at 0.5 μL min^−1^ for 15 min, followed by 25 μL of antibody at 3.5 μg mL^−1^ in MES buffer at 10 mM pH 6.5. The chip was left incubating overnight at 4 °C. The crosslinking reaction was stopped by washing three times with 20 mM PBS buffer at pH 7.4. 25 μL of skimmed milk as blocking agent at 0.5% (w/v) with 0.05% (w/v) Tween 20, were added and incubated for 30 min at 25 °C. Afterwards, 25 μL of the sample with the antigen were incubated for 10 min at 25 °C. Between each step, TO-RCH was washed three times with 20 mM PBS buffer at pH 7.4, after which the washing solution was completely removed. The SERS-based sandwich immunoassay was finalised by incubation of 25 μL at 1 nM of SERS immunotags for 10 min at 25 °C and washing with buffer three times. The antibody immobilisation and blocking were performed in a direction perpendicular to the detection zone, while the insertion of the antigen and SERS immunotags were parallel to the detection zone.

#### Characterisation of SERS-based microfluidic immunoassay

The biosensor was characterised for reproducibility, selectivity towards the antigen, reuse through regeneration, time-stability, and multiplexing. The procedure for the SERS-based immunoassay was performed as previously described,^23^ with the following modifications: for the selectivity assay, as a negative control, a non-infected RBCs culture was used and compared to an infected RBCs culture (containing *Pf*HRP2). For time stability studies, SERS-immunotags and RCH functionalised with anti-*Pf*HRP2 were kept in phosphate buffer pH 7.4 and PBS 10 mM at 4 °C during the assays; the regeneration study was accomplished by inserting 100 μL glycine-HCl pH 2.8 for 15 min in the SERS immuno-platform, and subsequently 1 μL of 1 M Tris-HCl pH 9 to restore the original pH of the solution and avoid denaturation of anti-*Pf*HRP2, followed by washing with PBS three times before repeating the incubation step with *Pf*HRP2 and the SERS-active SERS-immunotags. A multiplex assay was performed by adding SERS-immunotags with two different Raman reporters (MBA and DTNB) that recognise different antigens, namely, SERS-immunotags with MBA, which were formed with monoclonal anti-*Pf*HRP2 that recognises *Pf*HRP2, and SERS-immunotags with DTNB were formed with anti-peroxidase that recognises pLDH. The SERS-immunotags at 1 nM were incubated with the RCH immuno-platform loaded with equivalent amounts of anti-pLDH and anti-*Pf*HRP2 and with the respective antigens as described herein to allow the formation of the sandwich immunoassay.

##### Horseradish peroxidase enzymatic assay

To determine the viability of the immobilised antibodies on the capture platform, outside and inside the microfluidic chip, the binding of anti-peroxidase antibodies to peroxidase antigens was tested *via* a horseradish peroxidase enzymatic activity assay, as previously described.^48^

##### Optical spectroscopy

All absorption spectra were recorded in a UV-Vis spectrophotometer Cary 50 Bio (Varian®, Agilent, USA) using quartz cells with 1 cm path length (Hellma®), at room temperature. The absorbance at 405 nm for the enzymatic assays performed on the RCH off- and on-chip was measured in a multifunctional microplate reader TECAN SPARK 10M (Tecan Trading AG, Switzerland). The optical response of the polystyrene was measured with a double beam UV-VIS-NIR spectrometer (Lambda 950, PerkinElmer) equipped with an integrating sphere, in the wavelength range of 250–1100 nm.

##### Thermal material characterisation

Thermal material characterisation of RCH and PS was realised using thermogravimetric analysis (TGA) and differential scanning calorimetry (DSC) in nitrogen and in air. TGA-DTG analysis were carried out in a simultaneous thermal analyser (TGA-DSC—STA 449 F3 Jupiter) from Netzsch. The samples (10 mg for polystyrene and 6.1 mg for RCH) were loaded into a closed aluminium crucible and heated from room temperature to 700 at a rate of 10 °C min^−1^.

##### Morphological characterisation

SEM of the microfluidic chip was performed in a Hitachi™ 3030Plus tabletop workstation (Hitachi High-Tech Europe GmbH, Krefeld, Germany). Atomic force microscopy (AFM) analysis was performed in an Asylum Research MFP-3D Standalone AFM system (Oxford Instruments). AFM measurements were performed in AC mode in air. Silicon AFM probes (Olympus AC160TS, Olympus Corporation, Japan; *k* = 26 N m^−1^, *f*_0_ = 300 kHz) were used for AFM measurements. Static water contact angle measurements were performed with DataPhysics OCA 15 Plus, using 2 μL droplets of deionised water. The side view of the droplet was acquired by the system.

### Raman and SERS Measurements

Raman measurements were performed in a Renishaw inVia Qontor micro-Raman spectrometer equipped with an air-cooled charge-coupled device (CCD) as detector and a He–Ne laser operating at 32 mW of 632.81 nm. For the SERS-immunotags in solution (300 μL), and the final immunoassay performed on the membrane placed onto a microscope slide, the laser beam was focused with 5× (n.a. 0.12) and long-distance 50× (n.a. 0.5) respectively. An integration of 10 scans of 20 s each was used for all SERS-immunotags measurements. The intensity of the incident laser beam was 3.2 mW. Raman images of sandwich immunocomplexes on the RCH were obtained using a Raman point mapping method (scan of 21 × 21 μm, 1 μm steps). All spectra were obtained in triplicate, generating a total of 1233 points (pixels) per sample. Between different Raman sessions, the spectrograph was calibrated using the Raman line at 520.7 cm^−1^ of an internal crystalline Si sample. All SERS spectra were recorded at room temperature. All raw data were collected digitally with Wire 5.0 software. Noise reduction, available on the software, was used to estimate and remove the noise through principal component analysis (PCA). Baseline correction using a polynomial fitting (11^th^ order) was then performed, taking care to ensure minimal alteration of raw data.

### Statistical analysis

For enzymatic assays and SERS-activity assays, results were presented as mean ± standard-deviation from at least three independent experiments run in triplicates. Normality of the data distribution was assessed firstly by the Kolmogorov–Smirnov^49^ and then by Shapiro–Wilk^50^ test for increased statistical power. The antibody immobilisation efficiency and antigen detection in microfluidic assays were statistically compared using two-sample Student *t*-test, acquiring the *p*-value accordingly with Welch correction.^51^ To perform statistical group comparison tests, one-way analysis of variance (ANOVA) followed by the Tukey's multiple comparison test were applied.^52^ Outliers were identified by Grubbs test.^53^ Results were considered statistically significant when *p*-values were ≤0.05. Nonparametric analysis of variance (Kruskal–Wallis test^49^) was performed on the CLS score of individual immunoassay samples across all populations to test the statistical difference between groups. Results were considered statistically significant when *p*-values were ≤0.05. In the present case, this means that we only considered the results to be significant when the CLS score was sufficiently high to be considered as a result of specific binding, rather than non-specific binding of the SERS immunotags to the *Pf*HRP2.

## Conclusions

A novel microfluidic SERS immunoassay, based entirely on laser engraving of shrinkable PS sheets, was developed and its applicability to detect *Plasmodium* antigens evaluated. Shrinkable PS sheets were selected due to their optimal transparency (87% of transmittance at 633 nm) and ease of fabrication of the microfluidic chip. The final chip was obtained employing a laser power of 2.5 W and a velocity of 0.0381 m s^−1^, leading to a high-quality pattern, *i.e.*, with well-defined channels and without PS damage. Shrinking the bound PS sheets in an oven yielded smooth and hydrophilic channels of 129 ± 8 μm width, 998 ± 53 μm height and a detection chamber with a diameter of 559 ± 10 μm. The process resulted in a reduction of the device dimensions by 58% and allowed fabrication of a 3D multilayer chip in less than 30 min.

The system detected *Pf*HRP2 antigen in an iRBC culture supernatant at concentrations as low as 15.3 ± 0.2 pg mL^−1^, equivalent to approximately 0.0015% parasiteamia. Given the close relationship between plasma *Pf*HRP2 and total body parasite biomass, its detection may correlate with the severity of malaria disease in malaria-endemic areas.^54^ Furthermore, using iRBC culture supernatants, a linear range of 0.0001–0.0025% parasitaemia was measured, with a LOD of 0.0012 ± 0.0001% parasitaemia, equivalent to ≈69 parasites per μL.

Our multiplex approach can distinguish *Pf*HRP2 from *Pf*LDH, which opens the possibility of extending the detection to other febrile illnesses that tend to mimic malaria's clinical symptoms.^55^

Our combined approach of microfluidics and TO-RCH provides an excellent thermal stability over time, with its efficiency remaining at 90% of as prepared, after one week at 37 °C.

### Future scope and perspectives

The presented multilayer chip with an integrated TO-RCH platform represents an innovative method for the fabrication of microfluidic chips in a rapid way (≈hours). Moreover, the PS-based microfluidic scalable fabrication process makes it is more affordable than its PDMS-based microfluidic device counterparts, which require clean-room facilities and more complex protocols.^56^

Although clinical samples should be tested in the future, the seven-fold lower detection limits obtained suggest that our microfluidic SERS immunoassay is suitable for measurement of low-to-mid *Plasmodium* parasitaemia levels with minimal user handling and in less than 20 min. Moreover, High DCLS scores SERS signals are only obtained in the presence of the target antigen that allows the formation of the immunocomplex which showed an outstanding and reliably selective response towards the detection of *Plasmodium* antigens.

An ever-greater concern for malaria diagnosis is the ability of an assay not only to differentiate between *falciparum* and non-*falciparum* malaria, but also to respond to *pfhrp2* and *pfhrp3* deletions. Early diagnosis of these other febrile diseases will avoid unnecessary prescription of malaria medication, limiting the development of drug resistance.

Finally, the temperature stability of the SERS microfluidic immunoassay was evaluated, as it constitutes a requirement for diagnostic tests to be employed in POCT.^57^ Immunoassays usually require low temperature transport and storage, to prevent denaturation of the antibody.^33^

Our microfluidic chip fabrication methodology can be easily adapted to other immunoassays, providing a rapid, stable, and inexpensive multiplexed solution for immuno-sensing.

## Author contributions

M.J.O. conceptualisation, formal analysis, and writing – original draft. A.D. produced the *Pf*HRP2 recombinant protein, S.C. produced *P. falciparum* biological materials, F.S. helped to conceptualise the microfluidics device, T.R.C. supported AFM characterisation, H.A., R.F. and H.J.B. supervised the work, and together with M.P. and E.P., Writing – review & editing until its final form. H.A., R.F., H.J.B., M.P., E.P., R.M. and E.F provided the funding, fabrication and characterisation facilities and reviewed the final version of the manuscript. The manuscript was written through contributions of all authors. All authors have given approval to the final version of the manuscript.

## Conflicts of interest

There are no conflicts to declare.

## Supplementary Material

AN-148-D3AN00971H-s001
