# Supplementary material for: A simple polystyrene microfluidic device for sensitive and accurate SERS-based detection of infection by malaria parasites
Source: Analyst. 2023 Jul 28;148(17):4053–63. doi: 10.1039/d3an00971h (PMC10440799; doi:10.1039/d3an00971h)
Supplement: AN-148-D3AN00971H-s001 [file AN-148-D3AN00971H-s001.pdf]

Electronic Supplementary Material (ESI) for Analyst.
This journal is © The Royal Society of Chemistry 2023

Electronic Supplementary Information

Simple polystyrene microfluidic device for sensitive and accurate SERS-based detection of infection by malaria parasites

Maria João Oliveira[a,b], Soraia Caetano[d], Ana Dalot[a,b], Filipe Sabino[a,b], Tomás R. Calmeiro[a], Elvira Fortunato[a], Rodrigo Martins[a], Eulália Pereira[c], Miguel Prudêncio[d], Hugh J. Byrne[e], Ricardo Franco*[b] and Hugo Águas*[a]

[a]*CENIMAT-i3N, Departamento de Ciência dos Materiais, Faculdade de Ciências e Tecnologia, FCT, Universidade Nova de Lisboa, and CEMOP/UNINOVA, 2829-516 Caparica, Portugal.*

[b]*Associate Laboratory i4HB – Institute for Health and Bioeconomy, Faculdade de Ciências e Tecnologia, Universidade NOVA de Lisboa, 2829-516 Caparica, Portugal, and UCIBIO - Applied Molecular Biosciences Unit, Departamento de Química, Faculdade de Ciências e Tecnologia, Universidade NOVA de Lisboa, 2829-516 Caparica, Portugal.*

[c]*LAQV, REQUIMTE, Departamento de Química e Bioquímica, Faculdade de Ciências, Universidade do Porto, Rua do Campo Alegre, s/n, 4169-007 Porto, Portugal.*

[d]*Instituto de Medicina Molecular João Lobo Antunes, Faculdade de Medicina, Universidade de Lisboa, Av. Prof. Egas Moniz, 1649-028 Lisbon, Portugal.*

[e]*FOCAS Research Institute, Technological University Dublin, Camden Street, Dublin 8, Ireland*

S1. Preparation of biological samples 2
1.1. Expression, purification, and biochemical characterisation of *Pf*HRP2 2
1.2. *Plasmodium falciparum* culture supernatant, infected RBC, and parasite lysate 3
1.3. Characterisation of *Pf*HRP2 by SDS-PAGE and Western blotting 4
S2. Preparation of gold nanostars and SERS-immunotags. 6
2.1. Gold nanoparticles synthesis and functionalisation. 6
2.2. SERS-immunotags with covalently attached antibodies. 7
2.3. Production of Regenerated Cellulose Hydrogel 7
S3. Polystyrene chip fabrication 7
S4. Hydrophobicity of the microchannels 10
S5. Analytical parameters of the immunoassay 10
5.1. Comparison with sensitivity of malaria detection methods 10
5.2. Reproducibility and selectivity 11
5.3. Time and thermal stability assay 13
S6. References 14

S1. Preparation of biological samples

1.1. Expression, purification, and biochemical characterisation of *Pf*HRP2

Expression and purification of the recombinant *Pf*HRP2 were performed as described by Ndonwi *et al.* [1] with minor modifications. *Escherichia coli* BL21 (DE3) was chosen as the host system, transformed with a *Pf*HRP2 sequence, followed by a 6-His tag in the pET 15b vector kindly given by Professor Daniel E. Goldberg (Washington University, USA) [2].

The following reagents were used: Glycerol, ampicillin, potassium phosphate salts, magnesium sulphate, glycine, tris(hydroxymethyl)-aminomethane, citric acid, 3,3,5,5 tetramethylbenzidine (TMB), β-mercaptoethanol, polyvinylidene fluoride membrane, and sodium chloride were purchased from Sigma-Aldrich, St. Louis, MO, USA. Yeast extract, tryptone, and sodium dodecyl sulphate (SDS), were purchased from Panreac AppliChem, Gatersleben, Germany. Isoproprγl β-d^{-1}-thiogalactopyranoside (IPTG), Ponceau S, and molecular weight NZYTech marker I was from NZYTech, Lisbon, Portugal. Imidazole, Coomassie Blue R-250, methanol, and ammonium persulphate (APS) were from Alfa Aesar – Thermo Fisher Scientific, Waltham, USA. Protease inhibitors (Mini EDTA-free) and DNase I were from Roche, Switzerland and Ni-NTA resin from Qiagen, Hilden, Germany. 30% Acrylamide/Bis solution, 37.5:1, tetramethyl-ethylenediamine (TEMED) was purchased from Bio-Rad, Lisbon, Portugal. Transparent PS sheets purchased from Vaessen Creative shrink sheets, China; Tygon Tubing Coil - 1/16 OD X 0.02"(500 \textmu m) and Blunt-end Luer Lock Syringe Needles from Darwin Microfluidics, France; and Hamilton® GASTIGHT® syringe, 1700 series, PTFE Luer Lock 1750TLL from Sigma-Aldrich, St. Louis, USA. All chemicals were of the highest purity available. Ultrapure water (18.2 MΩ·cm at 25 °C) was used for the preparation of all solutions, unless stated otherwise.

A sample of a vial with a seed bacterial starter culture solution stored in a cryoprotective medium with 50 % (v/v) Glycerol (Sigma Aldrich, St. Louis, MO, USA) at -80 °C was collected and allowed to grow overnight in Luria-Bertani (LB) medium (Yeast extract 5 $g \cdot L^{-1}$, Tryptone 10 $g \cdot L^{-1}$ and NaCl 10 $g \cdot L^{-1}$) and ampicillin 50 $mg \cdot mL^{-1}$ for 16 h at 37 ºC, 220 rpm. Following the growth, the 20 mL from the overnight culture was divided and transferred to 2 L of LB medium. The culture was incubated at 37 ºC, 225 rpm to an optical density (OD) of 0.6 at 600 nm. Then, the temperature was lowered to 20 ºC and 0.5 mM of isoproprγl β-d-1-thiogalactopyranoside (IPTG, NZYTech, Portugal) was added and incubated for 20 h at 180 rpm. This concentration ensures that expression of the recombinant protein does not start at a high rate, which could be detrimental to protein folding [3].

Following the 20 h, the bacteria were harvested by centrifugation for 15 min, 6000 rpm at 4 °C, in 0.5 L centrifuge tubes (Beckman Coulter) in the Centrifuge Beckman Coulter Avanti J26-XPI with a JA-10 rotor. The *pellets* were resuspended in a wash buffer (50 mM potassium phosphate buffer pH 7.5, 30 mM NaCl, 30 mM imidazole, Alfa Aesar – Thermo Fisher Scientific, Waltham, USA). One tablet per 10 mL of protease inhibitors (Mini EDTA-free, Roche,

Switzerland), 50 µL of DNase I 5 $mg \cdot mL^{-1}$ (Roche, Switzerland), and 50 mM of magnesium sulphate ($MgSO_4$, Sigma-Aldrich, St. Louis, MO, USA) were added to the suspension. The suspension was subjected to French pressure cell at 20,000 psi (French pressure cell press, Thermo Electron Corporation – Thermo Fisher Scientific, Waltham, USA) three times and centrifuged at 45000 rpm for 1 h at 4 ºC in an ultra-centrifuge Optima LE-80K. The pellet was discarded, the supernatant collected and, then passed by gravity flow over an activated Ni-NTA resin (Ni-NTA Agarose, Qiagen, Hilden, Germany) pre-equilibrated with binding buffer (50 mM phosphate buffer pH 7.5, 500 mM NaCl, 30 mM imidazole - Alfa Aesar – Thermo Fisher Scientific, Waltham, USA), to promote protein binding. 500 mM NaCl was used to avoid weak electrostatic interactions of contaminant proteins with the column, and 30 mM of imidazole was used to remove proteins weakly bound to the column. 1 mL of Ni-NTA agarose was used for every 250 mL of supernatant and mixed by gentle shaking at room temperature for 1 h.

The soluble fraction from the cell lysate was loaded into the column, and the flow through was collected. The column was washed with wash buffer until an absorbance of less than 0.1 at 280 nm could be detected. Subsequently, the protein was eluted with elution buffer (50 mM phosphate buffer at pH 7.5, 30 mM NaCl, 500 mM imidazole), whereby the imidazole competes for the binding of Ni-NTA resin with the histidine residues of proteins [4]. Eluates were collected until the absorbance at 280 nm was less than 0.1. The eluates were concentrated by centrifugation (Amicon Ultra-4 Centrifugal Filter Units, 30k, 4 mL). The concentrated protein was aliquoted in cryovials with 200 µL each and stored in liquid nitrogen until further use. Each fraction, including flow-through, washes, and elutes were tested for purity and molecular weight estimation of the *Pf*HRP2 present using Sodium Dodecyl Sulphate-Polyacrylamide Gel Electrophoresis (SDS-PAGE) and Western blot (see section S1-1.3., below). Protein determination was carried out by the Bicinchoninic Acid (BCA) method using a kit from Sigma-Aldrich, St. Louis, USA.

1.2. *Plasmodium falciparum* culture supernatant, infected RBC, and parasite lysate

P. falciparum parasite strain NF54 had been previously cryopreserved in a 57% glycerol solution and was cultured in fresh RBC at 5% haematocrit in *Plasmodium* blood stage culture medium: RPMI medium (RPMI 1640, GIBCO) supplemented with 5% albumax II, 5% Glucose, 1% (v/v) HEPES buffer (GIBCO), 5% Sodium bicarbonate (GIBCO), 5% Gentamycin, 5% Hypoxanthine and 5% L-glutamine with 3% O_2, 5 % CO_2, and balance with N_2 gas and incubated at 37 °C, as previously described [5]. Growth medium was replaced by fresh one each two days. Parasitaemia values were obtained from thin blood smear readings using Giemsa stain on a microscope slide.

To obtain the samples for the SERS-based immunoassay, 10 mL iRBC cultures were centrifuged at 6000 g for 5 min. The supernatant was harvested and stored in a sterile centrifuge tube at −20°C or −80°C for later use. The pellet was resuspended in 10 mL PBS with 0.15 % (w/v) saponin at 4°C, homogenised by vortexing for 15 s, incubated for 5 min on ice, and homogenised for an additional 15 s. To induce parasite lysis, 40 µL cold lysis buffer (10 mM Tris-HCl pH 7.5, 0.5 mM EDTA, 1 mM dithiothreitol, 1 mM phenylmethylsulfonyl fluoride and 0.2 % v/v Tween 20)

was mixed with 10 µL of the lysed iRBCs and incubated for 15 min. These suspensions were centrifuged for 15 min at 6000 g at 4 °C, and 1 mL of PBS was added to the pellet which was washed three times with PBS and stored at −20°C.

1.3. Characterisation of *Pf*HRP2 by SDS-PAGE and Western blotting

All material used for the SDS-PAGE and Western blotting was cleaned with ethanol 70% (w/v) and ultrapure water before use. Acrylamide resolving gels (30% Acrylamide/Bis Solution, 37.5:1, Biorad) at 12% (v/v) were chosen based on the molecular weight of *Pf* HRP2 (≈50 kDa) since this percentage offers a separation range of 20-120 kDa. The ratio between sample analysed and sample buffer was always 1:1. Molecular weight NZYTech markers were used, and the samples were incubated at 95°C for 5 min in a thermo-block (Biosan TS-100, Latvia) and subsequently centrifuged at 13000 g for 2 min. The electrophoresis was performed in Bio-Rad Mini-PROTEAN Tetra Vertical Electrophoresis Cell at constant voltage (90 V) for 120 min. After electrophoresis, the SDS-PAGE gel was stained with 0.1% (w/v) Coomassie Blue R-250, 5% (v/v) glacial acetic acid, Scharlau, Spain, and 30% (v/v) methanol. Afterwards, the staining solution was removed and added the destaining solution (5% (v/v) glacial acetic acid and 30% (v/v) methanol) until the bands could be discerned and the gel background was clear.

For the Western blot protocol, an anti-PfHRP2 mouse antibody, produced in rabbit and conjugated with peroxidase was purchased from Sigma-Aldrich, St. Louis, MO, USA. After electrophoresis, the gels were blotted to a polyvinylidene fluoride membrane (Sigma-Aldrich, St. Louis, MO, USA). The blotting sandwich was prepared, and the inner module was placed into the electrophoresis chamber with transfer buffer pH 8.3 (Tris 25 mM, Glycine 192 mM, Methanol 20% (v/v)), a magnetic stir, and an ice pack. The transfer was run at 100 V for 60 min. Afterwards, the membrane was blocked for 60 min in 5% (w/v) skim milk in phosphate-buffered saline 1× and Tween 20 at 0.1% (w/v) (PBS-T, Sigma-Aldrich, St. Louis, MO, USA). The membrane was incubated for 60 min in anti-*Pf* HRP2 solution as the primary antibody (1:5000 dilution in 1% (w/v) skim milk in PBS-T) in a rocking shaker (mini-rocking shaker, Biosan, Latvia). Subsequently, the membrane was allowed to incubate for 60 min in the anti-mouse IgG–Peroxidase antibody solution as secondary antibody (1:10000 dilution in 1% (w/v) skim milk in PBS-T). Between each step, the membrane was washed 3 times with PBS-T for 5 min followed by washing with PBS at pH 7.4, 5 mM. A 3,3,5,5-tetramethylbenzidine solution (0.4 $g \cdot L^{-1}$) in citric acid buffer pH 5 at 25°C (Sigma-Aldrich, St. Louis, MO, USA) was allowed to react with the peroxidase linked to the membrane in the presence of 0.02% (v/v) hydrogen peroxide solution in the ratio 1:1 (Panreac AppliChem, Gatersleben, Germany). The gel images were further analysed using gel molecular weight analyser feature in the Origin Pro 2020 software.

The purified recombinant *Pf*HRP2 produced by *E. coli* BL21 (DE3) culture was evaluated by SDS-PAGE and revealed mainly two bands of approximately 67 kDa and 22 kDa (Figure S1a). The 67 kDa is a higher apparent molecular weight than the predicted molecular weight of *Pf*HRP2 (≈35 kDa) [6] but congruent with the ≈60 kDa from the purified native *Pf*HRP2 obtained herein from iRBCs culture (Fig. S1c), and by other authors [2,7]. The small increase in molecular weight (≈7 Da) has been also reported by other authors [1,8] and might be a consequence of the amino acid substitutions during the rapid overexpression of *Pf*HRP2 in *E. coli* leading to protein heterogeneity. Western-Blot with anti-*Pf*HRP2 confirmed the 67 kDa band as *Pf*HRP2 and the

reactivity of anti-*Pf*HRP2 monoclonal antibody towards its antigen (Fig. S1b). Thus, the recombinant *Pf*HRP2 expressed and purified from *E. coli* was suitable for the spiking studies.

Notwithstanding this, only one sequence of *Pf*HRP2 is encoded by its expression plasmid, and since *Pf*HRP2 presents geographical variability, testing only this copy would limit the reactivity on the immunoassay. Thus, the cultures of RBCs were used to identify native *Pf*HRP2 (Figs. S1c and d) to ensure that the possible variation of *Pf*HRP2 would not compromise the reactivity of the immunoassay. SDS-PAGE stained gel and Western-blot for the supernatants and lysed iRBCs allowed to assess the presence of native *Pf*HRP2 in both fractions which is in agreement with the observations reported by other authors [7].

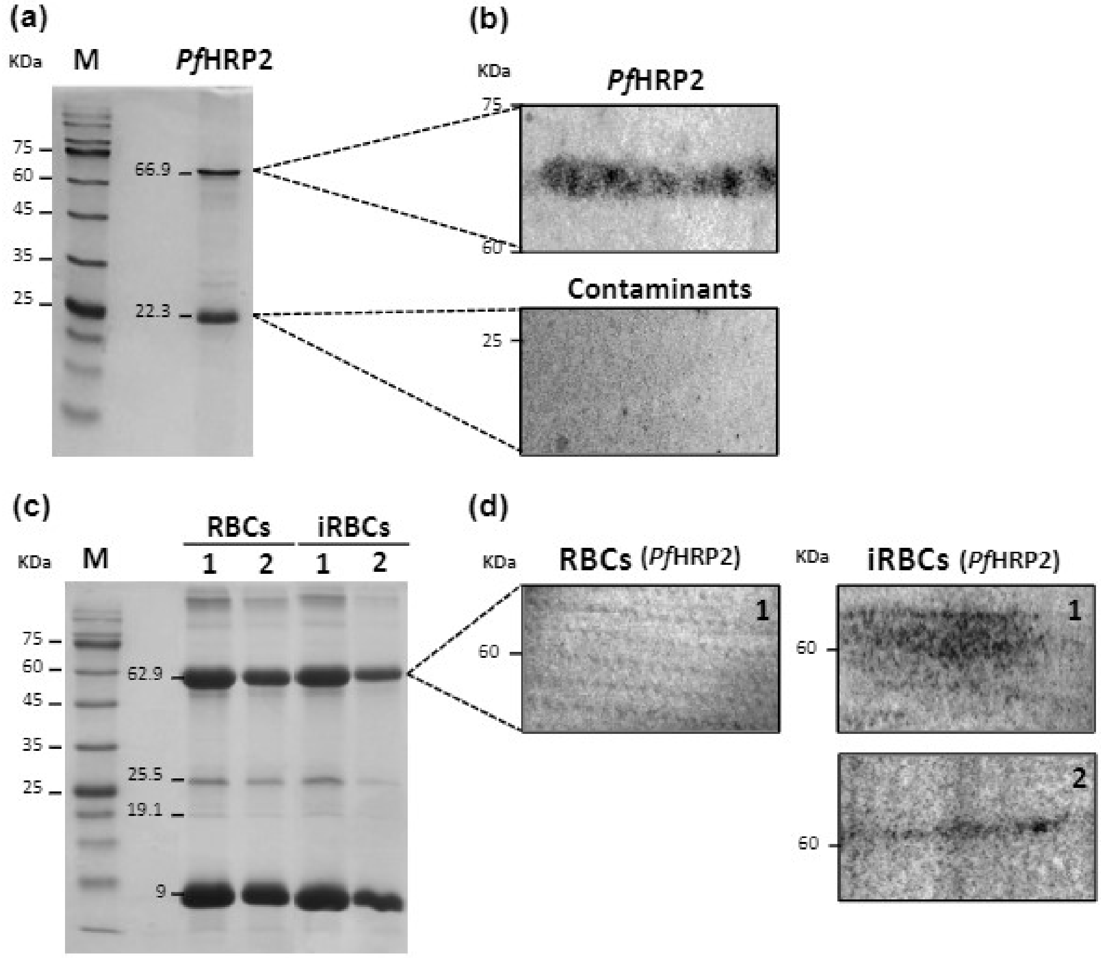

Figure S 1: Biochemical characterisation of *Pf*HRP2 purified from *E. coli* BL21 (DE3) culture and from RBC and iRBC cultures. (a) SDS - PAGE gel of the purified *Pf*HRP2 by a Ni-NTA affinity column. A molecular weight maker is shown in lane "M" and the second lane is the eluted sample from column purification, *i.e.*, *Pf*HRP2. (b) Western blot of the purified *Pf*HRP2 protein from the DE3 culture. (c) SDS - PAGE gel of the supernatant and lysed of infected and non-infected RBCs culture. A molecular weight maker is shown in lane "M". In the next four lanes, two correspond to non-infected RBCs culture and the last two to infected RBCs culture. The numbers "1" and "2" correspond to the lysed and supernatant samples, respectively. (d) Western blot of the iRBCs confirming the presence of *Pf*HRP2 in the lysed and supernatant samples. The same region was tested for non-infected RBCs culture as control.

S2. Preparation of gold nanostars and SERS-immunotags.

2.1. Gold nanoparticles synthesis and functionalisation.

The synthesis of gold spheres and star-shaped nanoparticles were performed according with the proposed methods of Ojea-Jiménez *et al.* [9] and Yuan *et al.* [10], respectively.

The synthesis started by immersing all glassware for the synthesis of NPs in freshly prepared *aqua regia*, based on a 1:3 mixture of nitric acid (Panreac AppliChem, Gatersleben, Germany), and hydrochloric acid (Fisher Chemical). Afterward, the glassware was vigorously washed with ultrapure water (18.2 MW.cm at 25 °C) prior to use. A variation of the conventional Turkevich synthesis protocol suggested by Ojea-Jiménez *et al.* [9] was applied. Briefly, 2 mL of a 343 mM trisodium citrate (Sigma-Aldrich, St. Louis, MO, USA) solution which were added to 98 mL of Milli-Q water in a round-bottom flask under heating and vigorous stirring (≈ 700 rpm) using a magnetic stirrer, whilst kept away from sunlight. After boiling, 69.2 µL of a 1.445 M $HAuCl_4$ solution (30 wt. % Au (III) chloride in dilute HCl, Sigma-Aldrich, St. Louis, MO, USA) were added to start the gold reduction reaction. After 5 min, the heating was stopped, and the suspension was cooled down to room temperature. Lastly, the suspension was transferred to a glass vial covered with aluminium foil and stored in the dark at 4°C until further use. After gold nanoparticles (AuNPs) synthesis and characterisation, this suspension was used as seed for AuNSs synthesis. AuNSs were synthesised using the seed-mediated growth method adapted from Yuan *et al.* [10]A solution of 15.5 µL of HAuCl4 at 1.445 M was added to 7 mL of a 2 nM suspension of AuNPs (diameter of 12 nm). Then, 450 µL of a 100 mM ascorbic acid (Fluka Buchs, Switzerland) solution and 450 µL of a 4 mM silver nitrate (Sigma-Aldrich, St. Louis, MO, USA) solution were added simultaneously. The resulting suspension was gently stirred for 30 s before centrifugation for 15 min at 3000 *g* (Centurion Scientific K3 Series centrifuge) and resuspension in 10 mL of ultrapure water. The diameter and concentration of the spherical nanoparticles were determined according to the relation described by Haiss *et al.* [11]. The same parameters for star-shaped nanoparticles were determined by the method of Puig *et al.* [12].

The functionalisation was performed as described in Oliveira *et al.* [13]. Gold nanostars were functionalised with a Raman reporter, 4-mercaptobenzoic acid (MBA, Sigma-Aldrich, St. Louis, MO, USA). The molecule works simultaneously as a capping agent for the AuNSs, a Raman reporter, and a bio-friendly intermediate for antibody bioconjugation. A suitable volume of a 10 mM ethanolic solution of MBA (ethanol, Sigma-Aldrich, St. Louis, MO, USA) was added to the AuNSs suspension under vigorous stirring. The solution was allowed to react overnight at room temperature to ensure a complete formation of a self-assembled monolayer. The excess of MBA was removed through centrifugation at 2500 *g* for 10 min, which was followed by redispersion. Complete monolayer formation was assessed by Agarose Gel Electrophoresis. The AuNSs suspension UV-Visible spectrum was checked to confirm successful functionalisation and to identify possible aggregation effects.

2.2. SERS-immunotags with covalently attached antibodies.

To promote crosslinking of the carboxylic acid groups of MBA or DTNB (MBA/DTNB) to the primary amines from the anti-HRP antibody molecules, several parameters were optimised, namely pH, reaction time, concentration and molar ratios of the reagents. The typical procedure

to produce AuNS–MBA/DTNB–anti-HRP SERS-immunotags was executed as follows: AuNS-MBA/DTNB – 0.2 nM - were washed by centrifugation at 2500 g at 4 °C for 10 min and resuspended in 5 mM MES buffer pH 6.5. A volume of 10 μL and 20 μL of EDC and SNHS at 1 mM was added to the colloidal suspension and let to react for 15 min in an orbital shaker (Biosan TS-100, Latvia) at 700 rpm at 25 °C. Following the carboxylic group activation, anti-HRP was added in appropriate amounts of antibody to obtain SERS-immunotags with a [AuNSs]:[anti-HRP] molar ratio of 1:422 [13]. The spent carbodiimide from AuNS–MBA/DTNB–anti-HRP, is removed through centrifugation. The incubation was also performed in an orbital shaker at 250 rpm, at 25 °C. Samples were then centrifuged for 10 min at 4 °C and 2500 g. The supernatant was discarded to remove excess protein and resuspended in 5 mM phosphate buffer pH 7.4. The blocking step was achieved through BSA to block non-specific interactions at the same molar ratio used for the anti-HRP. The AuNS–MBA/DTNB–anti-HRP–BSA conjugates, are referred as "SERS-immunotags". These SERS-immunotags were then used to incubate with HRP and/or anti-HRP to simulate the sandwich immunoassay. Incubation and wash steps were performed as done to form the SERS-immunotags.

2.3. Production of Regenerated Cellulose Hydrogel

Based on procedures reported in the literature for the dissolution of cellulose [14–16], the cellulose dissolution medium was prepared by mixing 4.6 wt.% LiOH, 15 wt%. urea in 80.4 wt.% deionised water. The solvent mixture was pre-cooled in a freezer at -25 °C, until it becomes a frozen solid. The frozen solution was then allowed to thaw at RT, and 6 wt.% of microcrystalline cellulose (MCC) (powder: 20 μm) was immediately added into the solvent system under vigorous stirring at -8 °C until its complete dissolution (≈ 30 min). A freezing-thawing cycle was performed to improve cellulose dissolution. Then, 5 mL of the resulting viscous solution was evenly spread on a glass plate (10×10 cm^2), and cellulose was regenerated for 60 min with glacial acetic acid. The sheet-like hydrogel membrane was thoroughly washed with deionised water to remove the remaining salts, dried at room temperature for 3 days and stored in air.

To immobilise antibodies, the RCHs were oxidised by using TEMPO/NaBr/NaClO system as described by Isogai *et al.* [17]. Firstly, 52 mL of ultrapure water were added to approximately 1 g of RCH. TEMPO and NaBr were dissolved and added to a final concentration of 2.5 mM and 15.28 mM, respectively. Then, 879.68 mM of NaClO were added in the solution and the pH was adjusted to 10 by adding 0.5 M NaOH. The TEMPO-oxidised RCHs (referred as "TO-RCH") were kept in the solution for 1 h under vigorous stirring (≈700 rpm). Finally, the TO-RCHs were thoroughly washed in ultrapure water and kept at room temperature in a solution of 10% (v/v) of H_2O_2.

S3. Polystyrene chip fabrication

The device was designed in a three-dimensional multi-layered system (Figure S2) that can be easily adjusted for other applications. In this design, the PS device is composed by three staked layers. The top layer was patterned with only the inlets and outlets. The middle layer is where

the immunoassay will be situated so is patterned with the channels, detection chambers and inlets and outlets. Finally, to seal the middle layer, a flat non-patterned PS sheet is placed as bottom layer. Between the middle and bottom layer, a TO-RCH membrane is placed to provide the surface for the SERS-based immunoassay. The fabrication procedure used throughout this work and its comparison with the conventional PDMS microfluidic chip fabrication are represented in Figure S2. Conventional PDMS microfabrication involves more complicated techniques than the combination of laser direct-writing micromachining in shrinkable PS sheets [18–21].

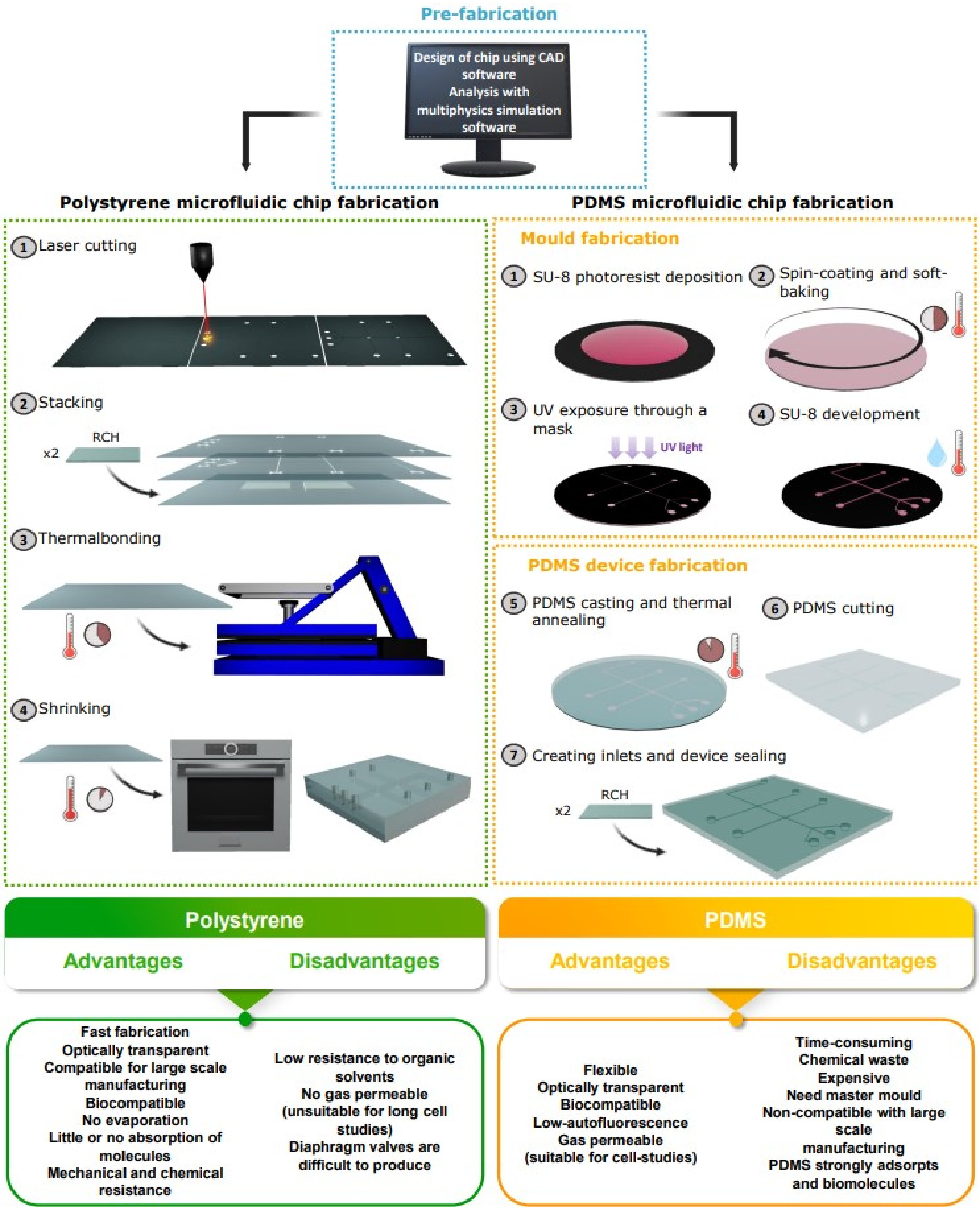

Figure S 2: Schematic illustrations of the fabrication process using PS or PDMS as the material of the chip. The pre-fabrication step is common to both fabrication processes. A chip design is drawn in CAD software and performance analyses can be performed in multiphysics simulation software. left: PS microfluidic chip fabrication process. (1) Laser cutting and engraving of PS substrates; (2) Stacking of the layers to form the 3D multilayer device; (3) Thermal treatment with pressure to bond the three layers of the chip; (4) Shrinking the device in an oven. right: (1) SU-8 photoresist application to Si wafer; (2) Spin-coating and soft baking to evaporate the solvent of the SU-8 photoresist; (3) Mask alignment and exposure to UV light; (4) SU-8 development, baking, and rinsing; (5) PDMS casting and thermal annealing; (6) PDMS chip cut and peeling-off; (7) Create inlets and sealing of the chip.

S4. Hydrophobicity of the microchannels

Hydrophobicity of a material is an important parameter when developing a device for biodiagnostic applications. The more hydrophobic a material is, the higher absorption of small hydrophobic molecules onto the channel wells [22,23]. This is one of the main problems of PDMS-based microfluidic devices. PS on the other hand, has little or no absorption of molecules [24]. The contact angles of PS before and after laser ablation, and after shrinking were measured to verify the surface wettability of the device. Before the laser treatment, PS had a contact angle of 70º ± 1 which agrees with the literature [19,23]. After the laser ablation, PS maintains the hydrophilicity giving a water contact angle of 68º ± 4. Additionally, after the thermal bonding, and shrinking processes, the hydrophilicity of PS increases to 64.3º ± 0.4, and 55º ± 3 which might be the result of a change in surface topography that decreases the surface roughness as can be seen by AFM measurements (Table S1). This is due to the re-solidification of PS after laser ablation and shrinking processes [19]. Thus, is possible to assume that the fabricated microchannels engraved in PS are hydrophilic.

Table S1: Surface roughness of PS throughout all the procedures of fabrication of the device of three samples of an area of $1\ \mu m^2$.

**Surface roughness of polystyrene (nm)**			
Unmodified	Laser ablation	Thermalbonding	Shrinking
1.48 ± 0.04	1.05 ± 0.03	0.95 ± 0.06	0.81 ± 0.03

S5. Analytical parameters of the immunoassay

5.1. Comparison with sensitivity of malaria detection methods

Table S2: Comparison of the sensitivity of analytical methods for malaria detection.

**Analytical method**	**Target**	**Type of sample**	**LOD** $(pg \cdot mL^{-1})$	**Ref.**
Microchannel capillary flow assay	*Pf*HRP2	*Pf*HRP2 spiked serum	8000	[25]
Droplet-based microfluidic device with antibody-immobilised magnetic beads	*Pf*HRP2	*Pf*HRP2 spiked human serum	2500	[26]
Gold screen-printed electrodes	*Pf*HRP2	*Pf*HRP2 spiked human serum	400	[27]
Q-Plex™ Human Malaria Array using dried blood spots	*Pf*HRP2	Patient blood	120	[28]
Carbon Nanofibers on Glass Microballoons Immunosensor	*Pf*HRP2	*Pf*HRP2 in PBS buffer	100	[29]
PS-based microfluidic SERS immunoassay ($\lambda_{Laser} =$ 633 nm)	*Pf*HRP2	*Pf*HRP2 spiked in iRBC culture supernatant	15	This work
**Analytical method**	**Target**	**Type of sample**	**LOD** $(parasites \cdot \mu L)$	**Ref.**
Silver nanospheres in SERS (mixed with blood lysate) ($\lambda_{Laser} =$ 633 nm)	Hemozoin	iRBC culture lysate	500	[30]
Silver nanospheres in SERS (synthetised within blood lysate) ($\lambda_{Laser} =$ 633 nm)	Hemozoin	iRBC culture lysate	220	[31]
Inductive sensor (impedance analysis)	Hemozoin	Hemozoin in PBS buffer	5	[32]
Gold nano-structure substrate ($\lambda_{Laser} =$ 532 nm)	Hemozoin	Patient blood lysates	0.1	[33]
Sandwich hybridization-based - LAMP	18S	Patient blood lysates	0.0013	[34]
PS-based microfluidic SERS immunoassay ($\lambda_{Laser} =$ 633 nm)	*Pf*HRP2	iRBC culture lysates<br>iRBC culture supernatant	750<br>69	This work

5.2. Reproducibility and selectivity

The ability of the device to discriminate *Pf*HRP2 within a complex sample can dictate its ability to avoid false positive results. Accordingly, the selectivity of the microfluidic SERS immunoassay was assessed by testing the responses to the RBCs and iRBCs in the presence or absence of *Pf*HRP2. When non-infected RBCs were assessed, the absence of the target antigen led to low coefficients (mean of 0.158 and 0.173 for the supernatant and lysate of RBCs, respectively), meaning a negative response (Figure 6a). High DCLS scores SERS signals are only obtained in the presence of the target antigen that allows the formation of the immunocomplex (Fig. S3). This behaviour was observed regardless of the type of sample (spiked or not with a purified sample of *Pf*HRP2), which proves that the microfluidic device enables a selective response towards *Pf*HRP2. In fact, these results are in agreement with PCR results reported by Jan *et al.* [28]. It should be mentioned that these samples also contain *Pf*HRP3, a protein closely related with *Pf*HRP2 with 85-90% homology in the nucleotide sequence, and therefore the possibility of some degree of cross-reactivity during the immunoassay cannot be excluded [35]. Yet, the microfluidic SERS immunoassay showed an outstanding and reliable selectivity response towards the detection of *Plasmodium* antigens.

The inter and intra-reproducibility of the SERS-based immunoassay were also evaluated by performing the immunoassay using three independent microfluidic devices. The relative standard deviation for the SERS signal between different devices was of 7.4% indicating similar responses across independent measurements. This can be considered as a good reproducibility as the obtained RSD is lower than 10% [36] (Figure S 3b).

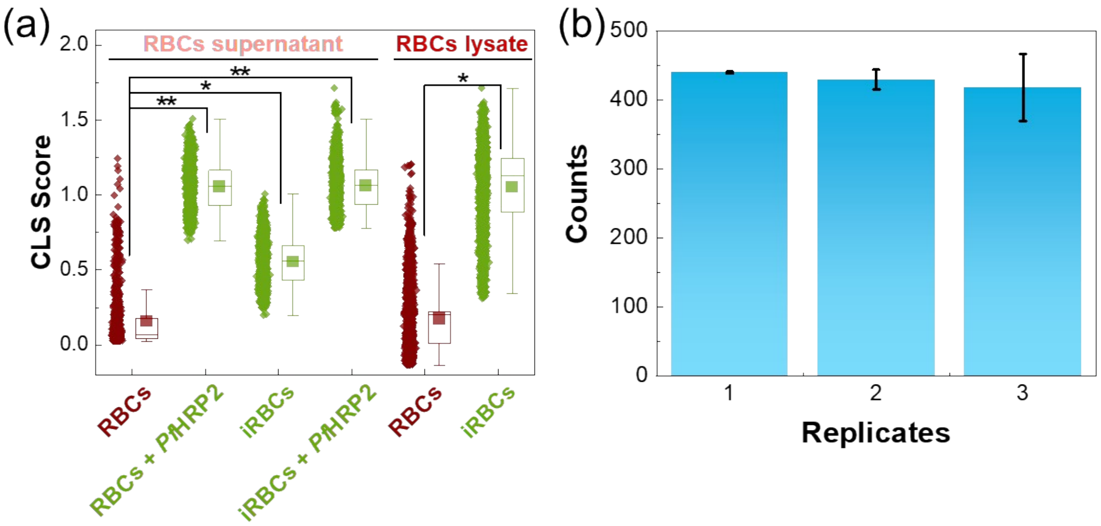

Figure S 3: Selectivity and reproducibility response of the microfluidic SERS immunoassay based on CLS results. (a) The CLS technique was used to quantify the contributions of a negative result (red data), obtained when using the supernatant and lysate of non-infected RBCs ("RBCs"). The positive results (green data) were obtained for the target antigen, *Pf*HRP2. The following samples were analysed: Supernatant samples – "RBCs" is the supernatant of non-infected RBCs; "RBCs + *Pf*HRP2" refers to using the supernatant of non-infected RBCs spiked with *Pf*HRP2; "iRBCs" corresponds to the supernatant of infected RBCs and "iRBCs + *Pf*HRP2" is the same supernatant with *Pf*HRP2; Lysate samples – "RBCs" is the lysate of non-infected RBCs and "iRBCs" is the lysate of infected RBCs. In the box plots, squares are the mean value for each distribution, the middle line represents the median and the top and bottom lines of each

box represent the 75 and 25 percentile values, respectively. Whiskers show upper and lower adjacent values which are 3 × IQR (interquartile range). Nonparametric analysis of variance by Kruskal–Wallis test (ANOVA) was performed for statistical comparisons. *p ≤ 0.05, **p ≤ 0.01. (b) Immunoassay activity over three independent chips with *Pf*HRP2 and SERS immunotags. Error bars correspond to the standard deviation from three independent measurements.

5.3. Time and thermal stability assay

The thermal stability of the microfluidic device activity was tested for iRBCs in devices stored at 37 ºC and compared to the activities observed from devices stored at 4 and 25ºC. The devices were evaluated 24 h after the antibody immobilisation was completed, and after 168 h (one week).

This type of capture platform with immobilised antibodies showed great stability after one week of storage at 4 ºC as previously described by Oliveira *et al.* [37]. This high stability was attributed in part to intermolecular cross-linking and to the high-water content environment provided by the RCH [37]. Similarly, as can be seen in Figure S4, the capture platform activity remained stable after one week at 4 and 25 ºC. For devices stored at 37 ºC, it is possible to observe a decrease in the SERS activity of 3% after 24 h and only 10% after one week. The high stability provided by the microfluidic device might be related to the low gas permeability of PS used as the material of the chip [22]. As a result, the PS encapsulates the capture platform allowing to minimise the evaporation of the buffer inside the microchannels. These results emphasise the importance of biomolecule stabilisation for achieving a high thermal stability suitable for application in malaria and other tropical infectious diseases.

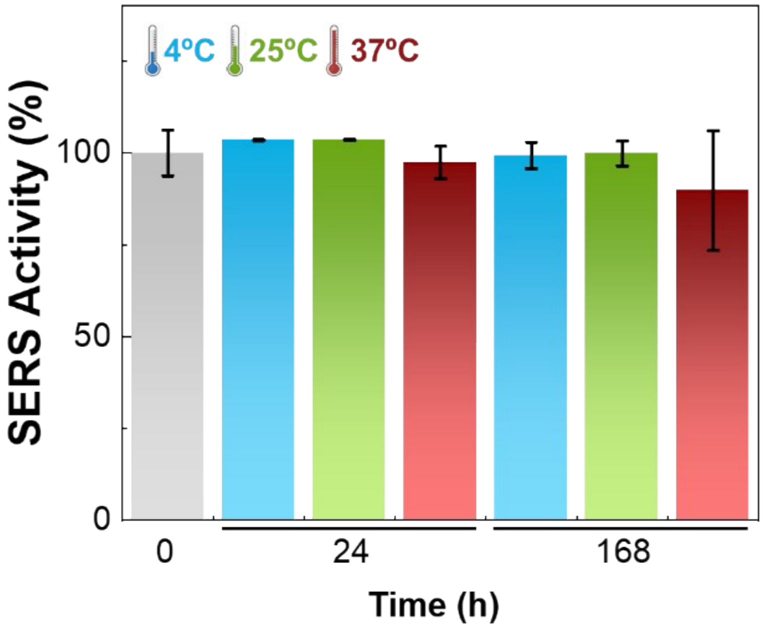

Figure S 4: Time and thermal stability study. The SERS activity was measured in three independent microfluidic devices at different temperatures, namely, 4, 25 and 37ºC after 24 h and 168 h. The grey bar represents the SERS activity

measured after the antibody immobilisation (t_0) and the results obtained for the 24 h and 168 h for the 4ºC (blue bars), 25ºC (green bars) and 37ºC (red bars) were compared to t_0.

S6. References

1 M. Ndonwi, O. O. Burlingame, A. S. Miller, D. M. Tollefsen, G. J. Broze and D. E. Goldberg, *Blood*, 2011, **117**, 6347–6354.
2 D. J. Sullivan, L. Y. Gluzman and D. E. Goldberg, *Science*, 1996, **271**, 219–222.
3 N. A. Burgess-Brown, Ed., *Heterologous Gene Expression in E.coli: Methods and Protocols*, Springer New York, New York, NY, 2017, vol. 1586.
4 A. Spriestersbach, J. Kubicek, F. Schäfer, H. Block and B. Maertens, in *Methods in Enzymology*, ed. J. R. Lorsch, Academic Press, New York, USA, 1st edn., 2015, vol. 559, pp. 1–15.
5 T. Ponnudurai, A. D. Leeuwenberg and J. H. Meuwissen, *Tropical and geographical medicine*, 1981, **33**, 50—54.
6 N. Hall, A. Pain, M. Berriman, C. Churcher, B. Harris, D. Harris, K. Mungall, S. Bowman, R. Atkin, S. Baker, A. Barron, K. Brooks, C. O. Buckee, C. Burrows, I. Cherevach, C. Chillingworth, T. Chillingworth, Z. Christodoulou, L. Clark, R. Clark, C. Corton, A. Cronin, R. Davies, P. Davis, P. Dear, F. Dearden, J. Doggett, T. Feltwell, A. Goble, I. Goodhead, R. Gwilliam, N. Hamlin, Z. Hance, D. Harper, H. Hauser, T. Hornsby, S. Holroyd, P. Horrocks, S. Humphray, K. Jagels, K. D. James, D. Johnson, A. Kerhornou, A. Knights, B. Konfortov, S. Kyes, N. Larke, D. Lawson, N. Lennard, A. Line, M. Maddison, J. McLean, P. Mooney, S. Moule, L. Murphy, K. Oliver, D. Ormond, C. Price, M. A. Quail, E. Rabbinowitsch, M.-A. Rajandream, S. Rutter, K. M. Rutherford, M. Sanders, M. Simmonds, K. Seeger, S. Sharp, R. Smith, R. Squares, S. Squares, K. Stevens, K. Taylor, A. Tivey, L. Unwin, S. Whitehead, J. Woodward, J. E. Sulston, A. Craig, C. Newbold and B. G. Barrell, *Nature*, 2002, **419**, 527–531.
7 B. Singh, J. N. McCaffery, A. Kong, Y. Ah, S. Wilson, S. Chatterjee, D. Tomar, M. Aidoo, V. Udhayakumar and E. Rogier, *Malar J*, 2021, **20**, 405.
8 E. L. Schneider and M. A. Marletta, *Biochemistry*, 2005, **44**, 979–986.
9 I. Ojea-Jiménez, N. G. Bastús and V. Puntes, *J. Phys. Chem. C*, 2011, **115**, 15752–15757.
10 H. Yuan, C. G. Khoury, H. Hwang, C. M. Wilson, G. A. Grant and T. Vo-Dinh, *Nanotechnology*, 2012, **23**, 075102.
11 W. Haiss, N. T. K. Thanh, J. Aveyard and D. G. Fernig, *Anal. Chem.*, 2007, **79**, 4215–4221.
12 X. Hu, Y. Dong, Q. He, H. Chen and Z. Zhu, *Journal of Chromatography B*, 2015, **990**, 96–103.
13 M. J. Oliveira, M. P. de Almeida, D. Nunes, E. Fortunato, R. Martins, E. Pereira, H. J. Byrne, H. Águas and R. Franco, *Nanomaterials*, 2019, **9**, 1561.
14 I. Cunha, R. Barras, P. Grey, D. Gaspar, E. Fortunato, R. Martins and L. Pereira, *Adv. Funct. Mater.*, 2017, **27**, 1606755.
15 Q. Yang, H. Fukuzumi, T. Saito, A. Isogai and L. Zhang, *Biomacromolecules*, 2011, **12**, 2766–2771.
16 M. He, Y. Zhao, J. Duan, Z. Wang, Y. Chen and L. Zhang, *ACS Appl. Mater. Interfaces*, 2014, **6**, 1872–1878.
17 A. Isogai, T. Saito and H. Fukuzumi, *Nanoscale*, 2011, **3**, 71–85.
18 K. N. Han, C. A. Li and G. H. Seong, *Annual Rev. Anal. Chem.*, 2013, **6**, 119–141.
19 I. Bilican and M. Tahsin Guler, *Applied Surface Science*, 2020, **534**, 147642.
20 C.-S. Chen, D. N. Breslauer, J. I. Luna, A. Grimes, W. Chin, L. P. Lee and M. Khine, *Lab Chip*, 2008, **8**, 622.

21 A. Grimes, D. N. Breslauer, M. Long, J. Pegan, L. P. Lee and M. Khine, *Lab Chip*, 2008, **8**, 170–172.
22 K. Ren, J. Zhou and H. Wu, *Acc. Chem. Res.*, 2013, **46**, 2396–2406.
23 B. H. Weigl, R. L. Bardell and C. Cabrera, in *Handbook of Biosensors and Biochips*, eds. R. S. Marks, D. C. Cullen, I. Karube, C. R. Lowe and H. H. Weetall, John Wiley & Sons, Ltd, Chichester, UK, 2008, p. hbb075.
24 S. Halldorsson, E. Lucumi, R. Gómez-Sjöberg and R. M. T. Fleming, *Biosensors and Bioelectronics*, 2015, **63**, 218–231.
25 S. Ghosh, K. Aggarwal, V. T. U., T. Nguyen, J. Han and C. H. Ahn, *Microsyst Nanoeng*, 2020, **6**, 5.
26 C. Kim, G. Hoffmann and P. C. Searson, *ACS Sens.*, 2017, **2**, 766–772.
27 A. Hemben, J. Ashley and I. Tothill, *Biosensors*, 2017, **7**, 28.
28 I. K. Jang, S. Aranda, R. Barney, A. Rashid, M. Helwany, J. C. Rek, E. Arinaitwe, H. Adrama, M. Murphy, M. Imwong, S. Proux, W. Haohankhunnatham, X. C. Ding, F. Nosten, B. Greenhouse, D. Gamboa and G. J. Domingo, *J Parasit Dis*, 2021, **45**, 479–489.
29 E. Gikunoo, A. Abera and E. Woldesenbet, *Sensors*, 2014, **14**, 14686–14699.
30 K. Chen, C. Yuen, Y. Aniweh, P. Preiser and Q. Liu, *Sci Rep*, 2016, **6**, 20177.
31 K. Chen, C. Perlaki, A. Xiong, P. Preiser and Q. Liu, *IEEE Journal of Selected Topics in Quantum Electronics*, 2016, **22**, 179–187.
32 A. P. Hole and V. Pulijala, *IEEE Sensors J.*, 2021, **21**, 1609–1615.
33 W. Wang, R. Dong, D. Gu, J. He, P. Yi, S.-K. Kong, H.-P. Ho, J. F.-C. Loo, W. Wang and Q. Wang, *Advances in Medical Sciences*, 2020, **65**, 86–92.
34 H. Pian, M. Yang, X. Sun and Z. Zheng, *Sensors and Actuators B: Chemical*, 2022, **365**, 131973.
35 K. E. Poti, D. J. Sullivan, A. M. Dondorp and C. J. Woodrow, *Trends in Parasitology*, 2020, **36**, 112–126.
36 C. Justino, A. Duarte and T. Rocha-Santos, *Sensors*, 2017, **17**, 2918.
37 M. J. Oliveira, I. Cunha, M. P. de Almeida, T. Calmeiro, E. Fortunato, R. Martins, L. Pereira, H. J. Byrne, E. Pereira, H. Águas and R. Franco, *J. Mater. Chem. B*, 2021, **9**, 7516–7529.
